# Assessment of agitation and aggression in inpatients with alcohol use disorder: A systematic review of informant‐based scales

**DOI:** 10.1111/acer.70138

**Published:** 2025-08-13

**Authors:** Willem S. Eikelboom, Denice S. A. M. Verberkt, Yvonne C. M. Rensen, Gwenny T. L. Janssen, Roy P. C. Kessels

**Affiliations:** ^1^ Centre of Excellence for Korsakoff and Alcohol‐Related Cognitive Disorders Vincent van Gogh Institute for Psychiatry Venray The Netherlands; ^2^ Donders Institute for Brain, Cognition and Behaviour Radboud University Nijmegen The Netherlands; ^3^ Tactus Addiction Care Deventer The Netherlands; ^4^ Klimmendaal Rehabilitation Centre Arnhem The Netherlands

**Keywords:** aggression, agitation, alcohol use disorder, assessment, irritability

## Abstract

**Background:**

Agitation and aggression are commonly observed in inpatients with alcohol use disorder (AUD). Adequate assessment is essential to provide appropriate care for these behaviors. To date, a systematic evaluation of existing measurement scales for use in AUD is lacking. The aim of this systematic review was to provide an overview of existing informant‐based scales to assess agitation and/or aggression and to evaluate their psychometric properties and applicability in people with AUD.

**Methods:**

Existing reviews on the assessment of agitation and/or aggression in psychiatric populations and neurocognitive disorders were searched to identify existing instruments to assess agitation and aggression. Next, for each scale, systematic literature searches were conducted in Embase, MEDLINE, and PsychINFO to evaluate the use in AUD and to identify psychometric studies using a validated methodological search filter. We applied the COnsensus‐based Standards for the selection of health Measurement INstruments (COSMIN) to evaluate the psychometric properties.

**Results:**

We included 20 unique scales for which we identified 86 studies reporting on psychometric properties and 65 studies that used any of these scales in AUD. To assess agitation and aggression retrospectively, the Rating Scale for Aggressive Behavior in the Elderly (RAGE) and Cohen‐Mansfield Agitation Inventory (CMAI) have the best psychometric qualities. The Pittsburgh Agitation Scale (PAS) showed the best psychometric properties of all scales that assess agitation and aggression during a prospective observation period. To assess agitation and aggression following an incident, the Overt Aggression Scale (OAS) has the best psychometric properties. Sixty‐five studies used any of the included scales to assess agitation and/or aggression in individuals with AUD, with the MOAS and OAS as the most commonly used scales within the AUD population.

**Conclusions:**

This review is the first to provide an overview of existing scales to assess agitation and aggression together with their psychometric properties and use in AUD. Findings guide clinicians and researchers to select the most appropriate instruments to improve the diagnosis and treatment of agitation and aggression in AUD.

## Graphical abstract

This systematic review provides an overview of existing scales to assess agitation and aggression in inpatients with alcohol use disorder (AUD), evaluating their psychometric properties and use in AUD populations. While numerous scales were identified, most have insufficient psychometric qualities. However, we identified the best available tools for retrospective assessment, prospective observation, or post‐incident evaluation. These findings help clinicians and researchers select appropriate instruments to improve diagnosis and treatment of agitation and aggression in AUD patients.

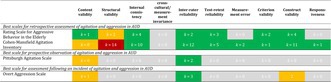

## INTRODUCTION

Agitation is characterized by feelings of restlessness, excessive motor activity, irritability, and increased responsiveness to internal and external stimuli (Garriga et al., 2016; Lindenmayer, 2000; Volicer et al., 2017). Some definitions of agitation incorporate aggression as the most extreme form of agitation (Sano et al., 2024; Zeller & Rhoades, 2010). Aggressive behaviors may include insulting or threatening others, harming oneself, damaging objects, or hurting others (Yudofsky et al., 1986). There is evidence for a sequence of agitated behavior progressing to verbal and physical aggression (Crowner et al., 2005; Hankin et al., 2011; Powell et al., 1994). However, other research has shown that agitation and aggression should be considered as two distinct symptoms with different underlying etiologies and manifestations (Derks et al., 2024; Volicer & Galik, 2018). In this view, agitation is considered restlessness, excessive motor activity, irritability, and increased responsiveness behavior without any focus or intent, while aggression is provoked or unprovoked behavior intended to cause harm to self, objects, or others (Patel & Hope, 1992; Volicer & Galik, 2018). For this paper, we adhere to these definitions of agitation and aggression.

Both agitation and aggression are frequently observed in individuals with alcohol use disorder (AUD). These behaviors may occur during intoxication (Heinz et al., 2011) and during alcohol withdrawal (Mirijello et al., 2015). However, agitation and aggression are also commonly observed in individuals with AUD during inpatient treatment when abstinence has already been realized (Bulgari et al., 2018; Dack et al., 2013; Ghossoub et al., 2019; Levola et al., 2014). The latter is the focus of this review. A subgroup of individuals with AUD who are especially inclined to exhibit agitation and aggression is those with alcohol‐related cognitive disorders such as Korsakoff's syndrome (Arts et al., 2017; Gerridzen et al., 2017).

Mechanistically, prior research suggests that factors such as impulsivity and impaired decision‐making mediate the relationship between AUD and agitation and aggression (Fineberg et al., 2014; Lejuez et al., 2010), while deficits in social cognitive abilities found in individuals with AUD may also contribute to agitated and aggressive behaviors in this population (Bora & Zorlu, 2017). In clinical practice, agitation and aggression have a significant negative impact on care staff (Hankin et al., 2011), leading to increased job stress (Itzhaki et al., 2018; Pelto‐Piri et al., 2020), post‐traumatic stress disorder (Jacobowitz, 2013), and may even cause physical injury (Pelto‐Piri et al., 2020). In addition, these behaviors may also negatively affect the optimal treatment of individuals with AUD with, consequently, poor treatment outcomes (Koekkoek et al., 2006).

International guidelines recommend adequate assessment as the essential first step when managing agitation and aggression in neurocognitive disorders (Cummings et al., 2023; Reus et al., 2016) and psychiatric conditions including AUD (Garriga et al., 2016; Pompili et al., 2021; Vieta et al., 2017). Clinicians are advised to use these standardized rating scales to measure the frequency and severity of agitated and aggressive behaviors, as their own clinical impression of agitation and aggression may be prone to bias (Pompili et al., 2021). These rating scales are referred to as informant‐based scales. That is, clinicians act as informants to evaluate the agitated and aggressive behaviors through direct observation and/or history taking. Informant‐based scales can be used to summarize and quantify the frequency and/or severity of agitation and aggression retrospectively, for instance, in the last 7 days or 2 weeks. Also, rating scales may be used to standardize the observation of agitation and aggression during a prospective observation period. Finally, rating scales can be used to assess the frequency and/or severity of specific behaviors directly following an incident of agitated or aggressive behaviors.

Irrespective of the type of informant‐based rating scale and its use, it is imperative that these assessment scales obtain valid and reliable scores (Ravyts et al., 2021). For example, as different care staff (i.e., informants) may be involved in the care of inpatients with AUD, outcomes of these informant‐based rating scales need to be reliable across informants. Furthermore, to evaluate the potential efficacy of interventions targeting agitation and aggression in a specific individual with AUD, rating scales should be able to capture these effects. Thus, information on the psychometric properties of rating scales to assess agitation and aggression is vital for clinicians working with inpatients with AUD. Furthermore, the use of valid and reliable rating scales is also essential when studying the manifestation of agitation and aggression in AUD populations (i.e., its prevalence and predictors) and when establishing the efficacy of new interventions.

To date, several reviews on existing scales for the assessment of agitation and aggression have been conducted (e.g., Choi et al., 2020; Ravyts et al., 2021; Volicer et al., 2017; Zeller & Rhoades, 2010). Although these reviews are useful, there are several limitations. First, while several reviews lack a systematic literature search (e.g., Ravyts et al., 2021; Volicer et al., 2017), all existing reviews lack a systematic psychometric evaluation of the discussed instruments (Prinsen et al., 2016). In addition, these reviews focus on specific populations other than AUD, such as neurocognitive disorders including dementia (Choi et al., 2020) and acquired brain injury (Pouwels et al., 2019). The manifestation of agitation and aggression may differ between these populations. For example, excessive motor activity is the most common manifestation of agitation in individuals with acquired brain injury, while aggressive behaviors and irritability are less commonly observed (see Phyland et al., 2021 for a review). In dementia, a combination of both irritability, verbal aggression, and physical aggression, as well as rejection of care, are most frequently observed in residential care facilities (e.g., Choi et al., 2017). Although little research has been done on the specific manifestation of these behaviors in AUD, previous studies have shown that individuals with AUD can show aggression toward both oneself and others (Ghossoub et al., 2019; Harford et al., 2013). Therefore, we will provide an overview of existing informant‐based scales to assess agitation and aggression for use in AUD, which may inform clinicians to select proper instruments for this specific population. However, no rating scale exists that was originally developed for the assessment of agitation and/or aggression in AUD.

Therefore, the aims of this systematic review are to (1) identify existing instruments to measure agitation and aggression irrespective of clinical condition, (2) examine the feasibility and psychometric properties for each scale irrespective of clinical condition, and (3) investigate the use of these instruments in inpatients with AUD.

## MATERIALS AND METHODS

This review was preregistered with PROSPERO (CRD42023431284) and conducted conform to the PRISMA guidelines (Appendix S1).

### Study selection

Study selection took place in two phases. First, we searched for existing reviews on the assessment of agitation and/or aggression in psychiatric populations and neurocognitive disorders to identify existing instruments to assess agitation and aggression. Second, once existing instruments were identified, we searched for studies that reported on psychometric properties and studies that were conducted in AUD populations for each identified instrument using two separate search strategies. Two independent authors (W.S.E., D.S.A.M.V.) screened titles and abstracts and subsequently inspected full texts for eligibility. In case of discrepancies, consensus was reached with the help of a third researcher (R.P.C.K.).

### Search strategy phase one

Existing reviews on the assessment of agitation and/or aggression were identified using a combination of search clusters “agitation or aggression,” and “psychiatry or neurocognitive disorders,” and “review” in MEDLINE, PsychINFO, and EMBASE (See Appendix S1). Reference lists of identified reviews were manually checked for potential other reviews of interest.

### Instrument selection

Once existing reviews were identified, we included instruments that were reported in these reviews based on the following criteria: the instrument (A) measures agitation and/or aggression, (B) is an informant‐based scale, (C) was developed for use in psychiatric populations, acquired brain injury, or neurocognitive disorders, (D) is not merely a subscale of a composite scale (e.g., the Neuropsychiatric Inventory, Brief Psychiatric Rating Scale), (E) is not an instrument used to predict future agitated or aggressive behaviors (e.g., The McNiel‐Binder Violence Screening Checklist, Brøset Violence Checklist), and (F) targets adults in an inpatient setting.

All included scales were categorized into (1) scales that assess agitation and/or aggression retrospectively, (2) scales that assess agitation and/or aggression during a prospective observation period, and (3) scales that assess agitation and/or aggression following an incident of these behaviors.

### Search strategy phase two

Next, we conducted two systematic literature searches in MEDLINE, PsychINFO, and EMBASE. First, we searched for studies on psychometric properties using search terms that included the names of each included scale (e.g., “Pittsburgh Agitation Scale”) together with a search filter developed to identify studies on measurement properties (Mokkink et al., 2024). Second, we searched for studies that used the included scales in an AUD sample, combining search terms that included the names of each included scale (e.g., “Pittsburgh Agitation Scale”) with a cluster of terms related to AUD (e.g., “alcohol,” “alcohol use disorder,” “alcoholism,” “Korsakoff”). The search strategies for both searches can be found in the Appendix S1.

After deduplication, titles and abstracts were screened for studies that reported on psychometric properties and studies that were conducted in AUD. The literature searches were conducted in June 2024 and updated in January 2025.

### Data synthesis

Psychometric qualities were evaluated using predefined standards following the COnsensus‐based Standards for the selection of health Measurement INstruments (COSMIN) (Mokkink et al., 2024; Prinsen et al., 2016). These guidelines provide consensus‐based definitions of measurement properties and an order of its importance in evaluating the quality of an outcome scale (Mokkink et al., 2024). For each scale, individual studies were evaluated using the predefined criteria described below. Next, scales were considered to have sufficient psychometric qualities if 75% of individual studies met these criteria (Mokkink et al., 2024; Prinsen et al., 2016).

Content validity is considered to be most important and entails the degree to which the content of an instrument reflects the construct being measured, that is, agitation and aggression (Mokkink et al., 2024). For this review, content validity was evaluated based on development studies (i.e., studies that reported on the development of a new scale) and content validity studies, if available. Also, authors W.S.E. and D.S.A.M.V. rated the relevance (i.e., all items are relevant for construct and target population), comprehensiveness (i.e., no key aspects of the construct are missing), and comprehensibility (i.e., all items are understood by the rater) of each scale for use in inpatients with AUD following the COSMIN procedure (Mokkink et al., 2024). Scales were considered to be comprehensive when they aimed to measure both agitation, verbal aggression, and physical aggression.

Next, the internal structure of a scale is considered important. Structural validity was evaluated using predefined COSMIN criteria (Mokkink et al., 2024). For example, studies that conducted an explanatory factor analysis were considered sufficient if they reported factor loadings of each item on its factor ≥0.30, a maximum of 10% of the items having factor loadings ≥0.30 on multiple factors, and an explained variance ≥50% with a structure that is in line with the theory about the construct being measured. In addition, internal consistency was evaluated and Cronbach's alpha ≥0.70 was considered sufficient (Mokkink et al., 2024).

Finally, cross‐cultural/measurement invariance, reliability, measurement error, criterion validity, construct validity, and responsiveness are considered important. Cross‐cultural/measurement invariance refers to the extent to which different cultural groups with the same latent trait respond similarly to the items of a scale. Cross‐cultural/measurement invariance is sufficient if studies show no important differences in multiple group factors analysis. Both test–retest reliability and inter‐rater reliability were considered sufficient if the intraclass correlation coefficient (ICC) or weighted Kappa were ≥0.70. Measurement error was considered sufficient if the smallest detectable change was lower than the minimal important change using anchor‐based methods (Mokkink et al., 2023). For sufficient criterion validity, a correlation coefficient or area under the curve ≥0.70 with a gold standard was required. For sufficient construct validity, correlation coefficients with instruments aimed at measuring similar constructs should be ≥0.50, while correlation coefficients with instruments aimed at measuring other constructs should be ≤0.30. Finally, responsiveness was defined as the ability to detect change over time and was considered sufficient if studies investigating responsiveness show findings that are in line with predefined hypotheses (Mokkink et al., 2024).

## RESULTS

### Identification of existing instruments

The search strategy of phase one resulted in 10 unique reviews on the assessment of agitation and/or aggression in psychiatric conditions (Garriga et al., 2016; Nordstrom & Allen, 2007; Pompili et al., 2021; Volicer et al., 2017), neurocognitive disorders (Choi et al., 2020; Gitlin et al., 2014; Pouwels et al., 2019; van der Linde et al., 2014), and older adults (Ravyts et al., 2021; Zeller & Rhoades, 2010). These existing reviews were used to identify existing instruments and reported on a total of *n* = 56 unique informant‐based scales, of which *n* = 20 scales met our predefined criteria.

Following the COSMIN guidelines (Mokkink et al., 2024), we considered each version of a scale as a separate scale. For the Cohen‐Mansfield Agitation Inventory (CMAI), different versions were available including the Brief Agitation Rating Scale, CMAI Observation tool, and CMAI‐Short Form, which differ with respect to the number of items. Different versions of the Overt Aggression Scale (OAS) were available including the Overt Aggression Scale‐Modified for Neurorehabilitation and the Modified Overt Aggression Scale (MOAS). Finally, the Staff Observation Aggression Scale has been modified and was also included in the current review (Staff Observation Aggression Scale‐Revised). Scales that were identified but did not meet our criteria can be found in the Appendix S1. Twenty scales met our criteria (listed in Table 1).

**TABLE 1:** Characteristics of included scales to assess agitation and aggression.

Scale	No. items	Administration time	Frequency/Severity	Observation period	Target population	Available languages	Full copy available
Retrospective assessment of agitation/aggression							
Aggressive Behavior Scale	4	NR	Frequency	Prior 7 days	Nursing home residents	English	Can be purchased online
Agitated Behavior Scale	14	NR	Severity	Unspecified	Traumatic brain injury	English, German, Danish, Spanish	Freely available
Brief Agitation Rating Scale	10	NR	Frequency	Prior 2 weeks	Nursing home residents	English, Norwegian	Presented in the study
Cohen‐Mansfield Agitation Inventory	29	20 min^a^	Frequency	Prior 2 weeks	Nursing home residents	English, Chinese, Dutch, French, Japanese, Italian, Korean, Polish, Chinese‐Taiwanese, Turkish	Freely available
Cohen‐Mansfield Agitation Inventory Short Form	14	NR	Frequency	Prior 2 weeks	Nursing home residents	English, German, Chinese‐Taiwanese	Presented in the study
Modified Overt Aggression Scale	4	NR	Severity	Prior 7 days	Psychiatric inpatients	English, Chinese‐Taiwanese, French, German, Italian, Spanish, Persian	Freely available
Rating Scale for Aggressive Behavior in the Elderly	21	5 min	Frequency	Prior 3 day	Nursing home residents	English, Chinese‐Taiwanese, French	Freely available
Ryden Aggression Scale‐2	26	20 min^a^	Frequency	Prior day	Dementia	English	Not available
Prospective observation of agitation/aggression							
Agitation Calmness Evaluation Scale	1	NR	Severity	Clinical evaluation	Dementia	English, Russian, Romanian	Presented in the study
Clinical Global Impression Scale for Aggression	1	<5 min^a^	Severity	Clinical evaluation	Psychiatric inpatients	English, German	Presented in the study
Cohen‐Mansfield Agitation Inventory Observational tool	29	NR	Frequency	5 h	Nursing home residents	English	Freely available
Overt Agitation Severity Scale	12	<5 min^a^	Frequency	15 min	Psychiatric inpatients	English	Presented in the study
Pittsburgh Agitation Scale	4	<5 min^a^	Severity	1–8 h	Dementia	English	Presented in the study
Positive and Negative Syndrome Scale Excited Component	5	<5 min^a^	Severity	Clinical evaluation	Psychiatric inpatients	English, Spanish	Presented in the study
Resistiveness to Care Scale	13	NR	Frequency/ severity	5 min ADL	Dementia	English	Not available
Scale for Observation of Agitation in Persons with Dementia	7	NR	Frequency/ severity	5 min	Dementia	English, Korean	Not available
Assessment following an incident of agitation/aggression							
Overt Aggression Scale	4	<5 min^a^	Severity	NA	Psychiatric inpatients	English, Spanish	Presented in the study
Overt Aggression Scale‐Modified for Neurorehabilitation	4	NR	Severity	NA	Traumatic brain injury	English	Presented in the study
Staff Observation Aggression Scale	5	<5 min	Severity	NA	Psychiatric inpatients	English, Dutch, German, Swedish	Presented in the study
Staff Observation Aggression Scale‐Revised	6	<5 min	Severity	NA	Psychiatric inpatients	English, Danish, Dutch, Japanese, Norwegian	Freely available upon request

Abbreviations: NA, not applicable; NR, not reported.

^a^ As reported in Pompili et al. (2021).

### Feasibility of existing instruments

Administration time was reported for 10 of the 20 scales (Table 1). Most scales can be administered within 5 min or less, while the CMAI and Ryden Aggression Scale‐2 may take up to 20 min. For most scales, the full copy of the English version is freely available online or can be found in the development study (Table 1).

### Psychometric properties of existing instruments

**FIGURE 1:**
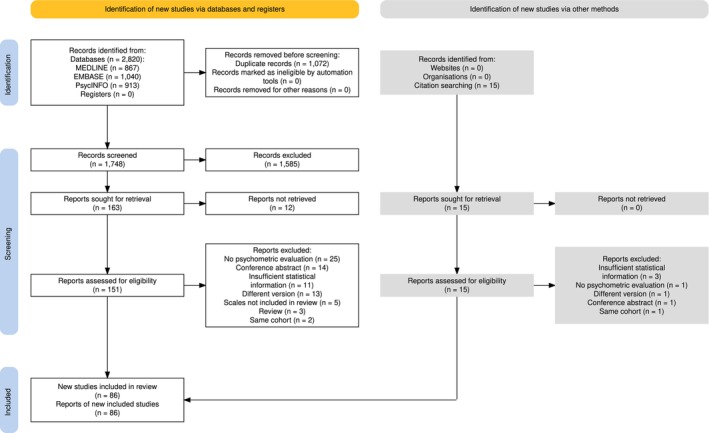
PRISMA flowchart of included studies reporting on psychometric properties of any of the included scales.

A total of 86 studies reported psychometric qualities for any of the 20 instruments (See Figure 1). Most studied informant‐based scales were the CMAI (*k* = 26), the MOAS (*k* = 10), the Agitated Behavior Scale (*k* = 8), the Rating Scale for Aggressive Behavior in the Elderly (RAGE) (*k* = 6), and the Staff Observation Aggression Scale‐Revised (k = 6). Construct validity (*k* = 45), inter‐rater reliability (*k* = 45), and internal consistency (*k* = 41) were most often studied, while content validity (*k* = 5), measurement error (*k* = 2), responsiveness (*k* = 1), and cross‐cultural/measurement invariance (*k* = 0) were hardly investigated across all scales.

The psychometric properties from the studies included in this review can be found in Table 2. Most included studies reported on the psychometric properties of scales that assess agitation and/or aggression retrospectively (*k* = 62), while fewer studies were included that reported on the psychometric properties of scales that assess agitation and/or aggression following an incident (*k* = 18), or of scales that assess agitation and/or aggression during a prospective observation period (*k* = 13).

**TABLE 2:** Use in AUD and psychometric evaluation of included scales to assess agitation and aggression.

Scale	Applied in AUD	No. psychometric studies	Content validity	Structural validity	Internal consistency	Cross‐cultural/measurement invariance	Inter‐rater reliability	Test–retest reliability	Measurement error	Criterion validity	Construct validity	Responsiveness
Retrospective assessment of agitation/aggression												
RAGE	0	6	1	2	4		2	3		2	4	
CMAI	5	26	0	14	10		12	5	2	1	11	1
ABSb	4	8	1	2	6		3				1	
MOAS	23	10	0	1	2		8	2		1	2	
BARS	1	4	0	1	4		2	2			3	
RAS‐2	0	1	0		1		1					
CMAI‐SF	0	3	1	3	3			1			2	
ABSa	0	4	0		5						2	
Prospective observation of agitation/aggression												
PAS	1	2	0				2					
CMAI‐O	0	1	0								1	
SOAPD	0	2	1	2	2		2	1			2	
PANSS‐EC	3	1	0	1	1		1				1	
OASS	2	2	0	1	1		2				1	
RTC	0	2	1	1	2						2	
CGI‐A	0	2	0							1	2	
ACES	0	1	0								1	
Assessment following an incident of agitation/aggression												
OAS	19	4	1				3				2	
OAS‐MNR	1	3	0				2				1	
SOAS	1	5	0				3				3	
SOAS‐R	6	6	0				2			1	4	

*Note*: 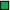 = sufficient; 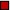 = insufficient; 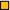 = mixed findings; 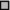 = not reported. The numbers in each cell represent the number of studies that reported on the psychometric properties and that were identified in this review. If no content validity study was conducted, content validity was evaluated by authors W.S.E. and D.S.A.M.V. following the COSMIN procedure (Mokkink et al., 2024).

Abbreviations: ABSa, Aggressive Behavior Scale; ABSb, Agitated Behavior Scale; ACES, Agitation Calmness Evaluation Scale; BARS, Brief Agitation Rating Scale; CGI‐A, Clinical Global Impression Scale for Aggression; CMAI, Cohen‐Mansfield Agitation Inventory; CMAI‐O, Cohen‐Mansfield Agitation Inventory Observational tool; CMAI‐SF, Cohen‐Mansfield Agitation Inventory Short Form; MOAS, Modified Overt Aggression Scale; OAS, Overt Aggression Scale; OAS‐MNR, Overt Aggression Scale‐Modified for Neurorehabilitation; OASS, Overt Agitation Severity Scale; PANSS‐EC, Positive and Negative Syndrome Scale Excited Component; PAS, Pittsburgh Agitation Scale; RAGE, Rating Scale for Aggressive Behavior in the Elderly; RAS‐2, Ryden Aggression Scale‐2; RTC, Resistiveness to Care Scale; SOAPD, Scale for Observation of Agitation in Persons with Dementia of the Alzheimer type; SOAS, Staff Observation Aggression Scale; SOAS‐R, Staff Observation Aggression Scale‐RevisedABSa, Aggressive Behavior Scale; ABSb, Agitated Behavior Scale; ACES, Agitation Calmness Evaluation Scale; BARS, Brief Agitation Rating Scale; CGI‐A, Clinical Global Impression Scale for Aggression; CMAI, Cohen‐Mansfield Agitation Inventory; CMAI‐O, Cohen‐Mansfield Agitation Inventory Observational tool; CMAI‐SF, Cohen‐Mansfield Agitation Inventory Short Form; MOAS, Modified Overt Aggression Scale; OAS, Overt Aggression Scale; OAS‐MNR, Overt Aggression Scale‐Modified for Neurorehabilitation; OASS, Overt Agitation Severity Scale; PANSS‐EC, Positive and Negative Syndrome Scale Excited Component; PAS, Pittsburgh Agitation Scale; RAGE, Rating Scale for Aggressive Behavior in the Elderly; RAS‐2, Ryden Aggression Scale‐2; RTC, Resistiveness to Care Scale; SOAPD, Scale for Observation of Agitation in Persons with Dementia of the Alzheimer type; SOAS, Staff Observation Aggression Scale; SOAS‐R, Staff Observation Aggression Scale‐Revised.

Of all scales that assess agitation and aggression retrospectively, the RAGE and the CMAI had the best psychometric qualities. The RAGE was the only scale with sufficient content validity in addition to sufficient internal consistency, construct validity, criterion validity, inter‐rater reliability, and test–retest reliability. The CMAI had mixed reports with respect to content validity, but was the only scale with sufficient measurement error and responsiveness, together with sufficient internal consistency, construct validity, criterion validity, inter‐rater reliability, and test–retest reliability (see Table 2).

The psychometric properties of scales that assess agitation and/or aggression during a prospective observation period were the best for the Pittsburgh Agitation Scale (PAS), although this scale had only mixed reports on content validity and sufficient inter‐rater reliability (Table 2). The remaining scales also had mixed or insufficient content validity and/or no reports of structural validity or internal consistency.

Of all scales that assess agitation and/or aggression following an incident of these behaviors, the OAS had the best psychometric properties, with sufficient inter‐rater reliability and mixed reports on content validity and construct validity (Table 2). The remaining scales also had mixed reports of content validity and had no or insufficient reports of structural validity, internal consistency, construct validity, and criterion validity.

### Use of existing instruments in AUD

**FIGURE 2:**
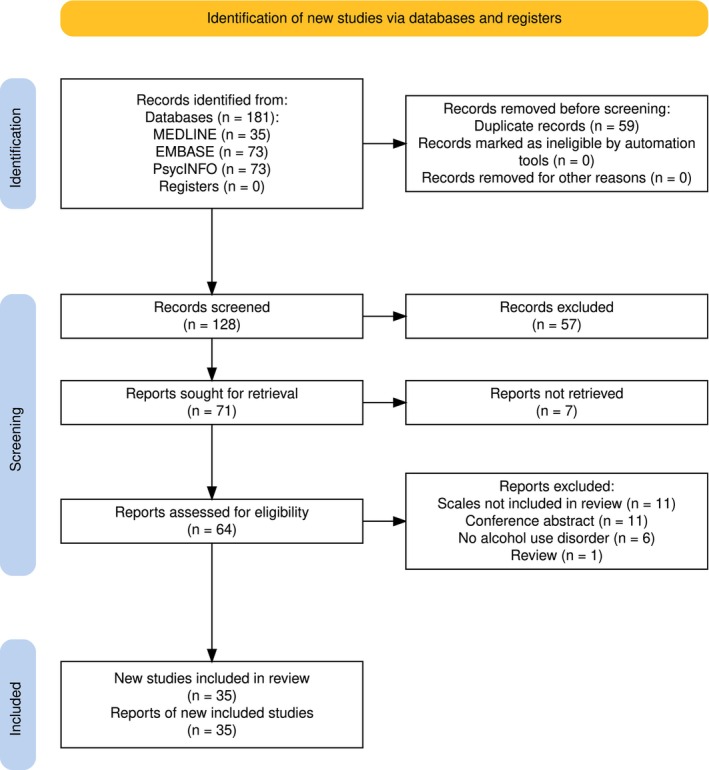
PRISMA flowchart of included studies that applied any of the included scales in patients with alcohol use disorder.

A total of 65 studies used any of the included scales to assess agitation and/or aggression in individuals with AUD (see Figure 2). No studies were found that used any of the included scales in patients with Korsakoff's syndrome, while one study applied the CMAI in patients with alcohol‐related dementia (Kunik et al., 2000). Of all scales that can be used to assess these behaviors retrospectively, the MOAS (*k* = 23) was most often used in AUD populations. Scales that assess these behaviors during a prospective observation period were rarely used in AUD, with the Positive and Negative Syndrome Scale Excited Component (*k* = 3) and Overt Agitation Severity Scale (*k* = 2) as the most commonly used in this population. Finally, of the scales that assess these behaviors following an incident, the OAS (*k* = 19) was most often used in AUD.

## DISCUSSION

We conducted a systematic review to evaluate the psychometric quality and use of existing informant‐based scales to assess agitation and/or aggression in inpatients with AUD. We identified 20 unique instruments that either assess agitation and/or aggression retrospectively, assess agitation and/or aggression during an observation period, or assess these behaviors following an incident. A total of 86 studies evaluated the psychometric quality of any of these scales, and 65 studies used any of the scales in individuals with AUD.

Although we were able to identify scales with the best psychometric properties for each category, the overall psychometric quality was insufficient for most scales. Although this important issue has been raised in previous reviews (e.g., Ravyts et al., 2021; van der Linde et al., 2014), to date no systematic evaluation of psychometric properties has been conducted on existing scales to measure agitation and aggression in psychiatric populations and neurocognitive disorders. Our findings show that most evidence is available for construct validity, inter‐rater reliability, internal consistency, and structural validity, while content validity, measurement error, responsiveness, and cross‐cultural/measurement invariance have been scarcely examined. This is problematic for the assessment of these behaviors in general, as well as for its application in AUD in specific.

First, unknown or insufficient content validity entails that items of current scales may not all be relevant to measure agitation and aggression, that relevant items are lacking, and/or that items may not be understood easily by care staff. While content validity was hardly evaluated in development studies and content validation studies, evaluation of content validity by the authors W.S.E. and D.S.A.M.V. following COSMIN guidelines also resulted in low ratings. This may be explained by the fact that most scales were not developed for use in psychiatric conditions such as AUD, but rather for use in neurocognitive disorders. In addition, responsiveness (i.e., the ability to detect change over time) and measurement error (i.e., error of an individual score not attributed to true change in the construct) were also hardly investigated. Only for the CMAI, anchor‐based minimal important change scores are available for use in neurocognitive disorders (Meunier et al., 2024). Consequently, it remains unknown whether changes in scores of the selected instruments over time reflect true changes in agitation and/or aggression. This is problematic as these instruments may be used to evaluate the effects of specific interventions to reduce agitation or aggression in an inpatient with AUD. In addition, these instruments may be used to study the course of agitation or aggression in AUD populations. Therefore, it is imperative to examine the responsiveness and measurement error in AUD populations of the best scales identified in this review. Finally, although the majority of scales have been translated into various languages using formal cross‐cultural adaptation procedures (e.g., Hellweg & Schuster‐Amft, 2016; Kim et al., 2020), no studies investigated cross‐cultural/measurement invariance in scores within a cross‐cultural sample. In the context of this review, cross‐cultural/measurement invariance refers to the degree to which scores on these translated scales remain valid, but also whether patients with similar levels of agitation obtain similar scores on included scales despite differences in cultural backgrounds, age, gender, or clinical conditions. This needs further evaluation as prior studies do suggest differences in the manifestation and management of agitation across ethnic minority groups with and without AUD as well as in different regions and cultures across the globe (e.g., Jivalagian et al., 2025; Wong et al., 2019). Moreover, this also touched upon the extent to which the manifestation of agitation observed in populations in which included scales were developed and tested, such as patients with neurocognitive disorders, is alike to AUD populations. Although studies have suggested differences in the clinical manifestation of agitation and aggression across various neurocognitive disorders (e.g., Choi et al., 2017; Phyland et al., 2021), research that directly compared the manifestation, frequency, and severity of agitation and aggression between neurocognitive disorders and AUD populations is lacking. Future studies are needed that investigate this in order to evaluate the measurement invariance properties of the included and recommended scales.

### Recommended scales to use in AUD

Of all scales that assess agitation and aggression retrospectively, the RAGE and the CMAI had the best psychometric qualities. The RAGE was developed and validated for use in older adults with various psychiatric conditions and/or neurocognitive disorders in an inpatient setting (Patel & Hope, 1992). The CMAI was originally developed for use in nursing home residents with a major neurocognitive disorder and is one of the most commonly used scales to assess neuropsychiatric symptoms such as agitation and aggression in long‐term care (Cohen‐Mansfield, 1986; Penko et al., 2021). The items of the RAGE are mostly related to irritability, rejection of care, verbal aggression, and physical aggression, while the CMAI mainly consists of items related to verbal aggression, physical aggression, and repetitive behaviors. Although the CMAI has been used in individuals with AUD in several studies, the RAGE has never been applied in AUD. Depending on the type of agitation‐related behaviors assessed, clinicians and researchers can choose to use either the RAGE or the CMAI in AUD populations.

The PAS had the best psychometric qualities of all identified scales that can be used to assess agitation and/or aggression during a prospective observation period. This scale allows clinicians and researchers to evaluate aberrant vocalization, excessive motor activity, aggression, and resisting care during an observation period of choice (Rosen et al., 1994). Although the PAS has been used in AUD populations, psychometric quality is poor with only sufficient inter‐rater reliability and mixed findings on the content validity.

The OAS had the best psychometric qualities of all identified scales that can be used to measure agitation and/or aggression after an incident. This scale measures primarily aggressive behaviors including verbal aggression, physical aggression against objects, physical aggression against oneself, and physical aggression against others, as well as potential interventions applied (Yudofsky et al., 1986). However, only the inter‐rater reliability has been found to be sufficient for the OAS, which shows that the overall psychometric quality is poor. The OAS has been used to assess aggression in AUD populations.

### Agitation and/or aggression

One complicated factor that hampers the assessment of agitation and aggression in AUD is the heterogeneity in terminology used to describe these behaviors (Choi et al., 2020; Pouwels et al., 2019; Volicer & Galik, 2018). This heterogeneity is also reflected in the names of the scales included in this study. Although several instruments only have “agitation” in their name (e.g., Agitated Behavior Scale, Cohen‐Mansfield Agitation Inventory), aggression has also been measured and vice versa (e.g., Rating Scale for Aggressive Behavior in the Elderly, Staff Observation Aggression Scale). Future studies are needed that address the nomenclature used to describe agitation and aggression in AUD.

### Strengths and limitations

Strengths of this study include the use of systematic literature searches and COSMIN guidelines to evaluate the psychometric properties of included scales. In addition, this review concludes with specific recommendations for the use of the best available scales to measure agitation and aggression in AUD populations specifically. However, there are also some limitations. First, only informant‐based scales were included, resulting in scales that measure observable behaviors related to agitation and aggression. Yet, these behaviors may also include mood swings and feelings of restlessness that may not be directly observed by care staff. However, only a few self‐report scales were identified in the existing reviews (e.g., Buss‐Perry Aggression Questionnaire, Buss‐Perry Aggression Questionnaire), with unknown psychometric quality. Furthermore, self‐reports of agitation and aggression may be hampered by memory impairments and reduced illness insight in alcohol‐related cognitive disorders (Arts et al., 2017; Walvoort et al., 2016). Second, content validity was evaluated by the authors (W.S.E. and D.S.A.M.V.) based on its relevance, comprehensiveness, and comprehensibility following the COSMIN procedure (Mokkink et al., 2024). This resulted in mixed findings for most scales, but for various reasons. It would have been insightful to differentiate between the relevance (i.e., all items are relevant for construct and target population), comprehensiveness (i.e., no key aspects of the construct should be missing), and comprehensibility (i.e., all items are understood by the rater) of the items in relation to agitation and aggression and the comprehensibility of the scale for use in AUD in inpatients. Finally, we found almost no studies that evaluated the responsiveness of scales. Still, included scales have been used frequently to evaluate the effectiveness of psychological or pharmacological interventions targeting agitation (e.g., Ramos et al., 2024). Yet, these studies did not explicitly mention the evaluation of responsiveness in their title/abstract and therefore were not captured in our systematic search. This may have led to an underestimating of the quality of responsiveness for the included scales.

### Recommendations for future research

Future studies should provide additional evidence of the psychometric quality of the selected scales. This review guides these research endeavors. For the RAGE, more research is needed to establish its structural validity, cross‐cultural/measurement invariance, measurement error, and responsiveness, together with the applicability in AUD. For the CMAI, future studies should investigate the content validity, structural validity, and cross‐cultural/measurement invariance. For both the PAS and OAS, more research is needed to establish the content validity, structural validity, internal consistency, cross‐cultural/measurement invariance, test–retest reliability, measurement error, criterion validity, construct validity, and responsiveness of these instruments.

Moreover, even though 65 studies have applied scales for assessing agitation and aggression in AUD, these patients had most likely no cognitive disorders, as these studies did not report on the cognitive status of these samples. The applicability and feasibility of these scales in AUD patients with mild or major neurocognitive disorder remain to be investigated.

## CONCLUSIONS

This review is the first to provide an overview of existing scales to assess agitation and aggression and their psychometric properties and use in AUD. Although our findings clearly show that there are numerous scales available to assess agitation and aggression, the psychometric qualities of these instruments are insufficient for most scales available. The findings of this review are relevant for clinicians and researchers who want to use specific scales to assess agitation and aggression retrospectively, during a prospective observation period, or following an incident.

## AUTHOR CONTRIBUTIONS

W.S.E., D.S.A.M.V., and R.P.C.K. designed the study. W.S.E. and D.S.A.M.V. conducted the literature search, study selection, and data extraction. W.S.E. analyzed the data and interpreted the data, assisted by D.S.A.M.V. W.S.E. drafted the first version of the manuscript, while D.S.A.M.V., Y.C.M.R., G.T.L.J., and K.P.C.K. critically reviewed the manuscript. All authors read and approved the final version of the manuscript.

## CONFLICT OF INTEREST STATEMENT

All authors declare no competing interests.

## Supporting information


Appendix S1

## Data Availability

The data that support the findings of this study are available from the corresponding author upon reasonable request.
