# Supplementary material for: Assessment of agitation and aggression in inpatients with alcohol use disorder: A systematic review of informant‐based scales
Source: Alcohol Clin Exp Res (Hoboken). 2025 Aug 13;49(9):1877–88. doi: 10.1111/acer.70138 (PMC12463754; doi:10.1111/acer.70138)
Supplement: Supplementary file 1 — Appendix S1 [file ACER-49-1877-s001.docx]

**Supplemental Material: PRISMA Checklist**

| **Section and Topic** | **Item #** | **Checklist item** | **Location where item is reported** |
| --- | --- | --- | --- |
| **TITLE** | | |  |
| Title | 1 | Identify the report as a systematic review. | Title |
| **ABSTRACT** | | |  |
| Abstract | 2 | See the PRISMA 2020 for Abstracts checklist. | Page 2 |
| **INTRODUCTION** | | |  |
| Rationale | 3 | Describe the rationale for the review in the context of existing knowledge. | Pages 3-4 |
| Objectives | 4 | Provide an explicit statement of the objective(s) or question(s) the review addresses. | Pages 4-5 |
| **METHODS** | | |  |
| Eligibility criteria | 5 | Specify the inclusion and exclusion criteria for the review and how studies were grouped for the syntheses. | Page 5 |
| Information sources | 6 | Specify all databases, registers, websites, organisations, reference lists and other sources searched or consulted to identify studies. Specify the date when each source was last searched or consulted. | Pages 5-6 |
| Search strategy | 7 | Present the full search strategies for all databases, registers and websites, including any filters and limits used. | Supplemental Materials |
| Selection process | 8 | Specify the methods used to decide whether a study met the inclusion criteria of the review, including how many reviewers screened each record and each report retrieved, whether they worked independently, and if applicable, details of automation tools used in the process. | Pages 5-6 |
| Data collection process | 9 | Specify the methods used to collect data from reports, including how many reviewers collected data from each report, whether they worked independently, any processes for obtaining or confirming data from study investigators, and if applicable, details of automation tools used in the process. | Pages 5-6 |
| Data items | 10a | List and define all outcomes for which data were sought. Specify whether all results that were compatible with each outcome domain in each study were sought (e.g. for all measures, time points, analyses), and if not, the methods used to decide which results to collect. | Pages 6-8 |
|  | 10b | List and define all other variables for which data were sought (e.g. participant and intervention characteristics, funding sources). Describe any assumptions made about any missing or unclear information. | Pages 6-8 |
| Study risk of bias assessment | 11 | Specify the methods used to assess risk of bias in the included studies, including details of the tool(s) used, how many reviewers assessed each study and whether they worked independently, and if applicable, details of automation tools used in the process. | Not applicable |
| Effect measures | 12 | Specify for each outcome the effect measure(s) (e.g. risk ratio, mean difference) used in the synthesis or presentation of results. | Not applicable |
| Synthesis methods | 13a | Describe the processes used to decide which studies were eligible for each synthesis (e.g. tabulating the study intervention characteristics and comparing against the planned groups for each synthesis (item #5)). | Pages 7-8 |
|  | 13b | Describe any methods required to prepare the data for presentation or synthesis, such as handling of missing summary statistics, or data conversions. | Pages 7-8 |
|  | 13c | Describe any methods used to tabulate or visually display results of individual studies and syntheses. | Not applicable |
|  | 13d | Describe any methods used to synthesize results and provide a rationale for the choice(s). If meta-analysis was performed, describe the model(s), method(s) to identify the presence and extent of statistical heterogeneity, and software package(s) used. | Pages 7-8 |
|  | 13e | Describe any methods used to explore possible causes of heterogeneity among study results (e.g. subgroup analysis, meta-regression). | Not applicable |
|  | 13f | Describe any sensitivity analyses conducted to assess robustness of the synthesized results. | Not applicable |
| Reporting bias assessment | 14 | Describe any methods used to assess risk of bias due to missing results in a synthesis (arising from reporting biases). | Not applicable |
| Certainty assessment | 15 | Describe any methods used to assess certainty (or confidence) in the body of evidence for an outcome. | Not applicable |
| **RESULTS** | | |  |
| Study selection | 16a | Describe the results of the search and selection process, from the number of records identified in the search to the number of studies included in the review, ideally using a flow diagram. | Page 8 |
|  | 16b | Cite studies that might appear to meet the inclusion criteria, but which were excluded, and explain why they were excluded. | Not applicable |
| Study characteristics | 17 | Cite each included study and present its characteristics. | Table 1, Table 2 |
| Risk of bias in studies | 18 | Present assessments of risk of bias for each included study. | Not applicable |
| Results of individual studies | 19 | For all outcomes, present, for each study: (a) summary statistics for each group (where appropriate) and (b) an effect estimate and its precision (e.g. confidence/credible interval), ideally using structured tables or plots. | Table 1, Table 2 |
| Results of syntheses | 20a | For each synthesis, briefly summarise the characteristics and risk of bias among contributing studies. | Pages 8-10 |
|  | 20b | Present results of all statistical syntheses conducted. If meta-analysis was done, present for each the summary estimate and its precision (e.g. confidence/credible interval) and measures of statistical heterogeneity. If comparing groups, describe the direction of the effect. | Table 2 |
|  | 20c | Present results of all investigations of possible causes of heterogeneity among study results. | Not applicable |
|  | 20d | Present results of all sensitivity analyses conducted to assess the robustness of the synthesized results. | Not applicable |
| Reporting biases | 21 | Present assessments of risk of bias due to missing results (arising from reporting biases) for each synthesis assessed. | Not applicable |
| Certainty of evidence | 22 | Present assessments of certainty (or confidence) in the body of evidence for each outcome assessed. | Not applicable |
| **DISCUSSION** | | |  |
| Discussion | 23a | Provide a general interpretation of the results in the context of other evidence. | Pages 10-11 |
|  | 23b | Discuss any limitations of the evidence included in the review. | Page 11 |
|  | 23c | Discuss any limitations of the review processes used. | Page 13 |
|  | 23d | Discuss implications of the results for practice, policy, and future research. | Pages 13-14 |
| **OTHER INFORMATION** | | |  |
| Registration and protocol | 24a | Provide registration information for the review, including register name and registration number, or state that the review was not registered. | Page 5 |
|  | 24b | Indicate where the review protocol can be accessed, or state that a protocol was not prepared. | Page 5 |
|  | 24c | Describe and explain any amendments to information provided at registration or in the protocol. | Not applicable |
| Support | 25 | Describe sources of financial or non-financial support for the review, and the role of the funders or sponsors in the review. | Page 14 |
| Competing interests | 26 | Declare any competing interests of review authors. | Page 15 |
| Availability of data, code and other materials | 27 | Report which of the following are publicly available and where they can be found: template data collection forms; data extracted from included studies; data used for all analyses; analytic code; any other materials used in the review. | Not applicable |

**Supplemental Material: Search strategy phase 1**

**To identify reviews on existing scales to assess agitation and/or aggression**

MEDLINE

1 Exp Psychomotor Agitation/ OR Exp Aggression/ OR (agitat* OR irritability OR aggress* OR disinhibit* OR motor activit*).ti,ab.

2 Exp brain diseases/ OR exp mental disorders/ OR (brain disease* OR brain disorder* OR mental disease* OR mental* disorder* OR mental ilIness* OR psychiatric disorder* OR neurocognit* disorder* OR dementia).ti,ab.

3 ((systematic review/ OR review/ OR meta-analysis/ OR review OR metaanaly* OR meta-anal* OR systematic search*) OR (systematic* ADJ3 literature)).ti,ab.

4 1 AND 2 AND 3

PsychINFO

1 exp agitation/ OR exp aggressive behavior/ OR exp aggressiveness/ OR (agitat* OR irritability OR aggress* OR disinhibit* OR motor activit*).ti,ab,id.

2 exp brain diseases/ OR exp mental disorders/ OR (brain disease* OR brain disorder* OR mental disease* OR mental* disorder* OR mental ilIness* OR psychiatric disorder* OR neurocognit* disorder* OR dementia).ti,ab,id.

3 (meta analysis OR systematic review OR literature review).md. OR (review OR metaanaly* OR meta-anal* OR systematic search* OR (systematic* ADJ3 literature)).ti,ab,id.

4 1 AND 2 AND 3

EMBASE

1 exp agitation assessment/ OR exp agitation/ OR exp aggression/ OR exp aggression assessment/ OR (agitat* OR irritability OR aggress* OR disinhibit* OR motor activit*).ti,ab.

2 exp brain diseases/ OR exp mental disorders/ OR exp central nervous system disease/ OR (brain disease* OR brain disorder* OR mental disease* OR mental* disorder* OR mental ilIness* OR psychiatric disorder* OR neurocognit* disorder* OR dementia).ti,ab.

3 review/ OR systematic review/ OR meta-analysis/ OR (review OR metaanaly* OR meta-anal* OR systematic search* OR (systematic* ADJ3 literature)).ti,ab

4 1 AND 2 AND 3

**Supplemental Material: Search strategy phase 2**

**To identify studies on psychometric properties**

*MEDLINE*

1 (Agitated Behavior Scale OR Agitated Behaviour Scale).ti,ab.

2 (Rating Scale for Aggressive Behavior in the Elderly OR Rating Scale for Aggressive Behaviour in the Elderly).ti,ab.

3 Staff Observation Aggression Scale.ti,ab.

4 (Aggressive Behavior Scale OR Aggressive Behaviour Scale).ti,ab.

5 Brief Agitation Rating Scale.ti,ab.

6 (Cohen-Mansfield Agitation Inventory Short Form OR Short Form Cohen-Mansfield Agitation Inventory OR CMAI-SF).ti,ab.

7 Overt Aggression Scale.ti,ab.

8 (Overt Aggression Scale-Modified for Neurorehabilitation OR OAS-MNR).ti,ab.

9 (Cohen-Mansfield Agitation Inventory OR Cohen-Mansfield Agitation OR CMAI).ti,ab.

10 (Clinical Global Impression Scale for Aggression OR CGI-A).ti,ab.

11 Overt Agitation Severity Scale.ti,ab.

12 Resistiveness to Care Scale.ti,ab.

13 (Scale for Observation of Agitation in Persons with Dementia of the Alzheimer type OR SOAPD).ti,ab.

14 (Staff Observation Aggression Scale-Revised OR Revised Staff Observation Aggression Scale OR Modified Observation Aggression Scale OR Adapted Observation Aggression Scale OR SOAS-R).ti,ab.

15 (Modified Overt Aggression Scale OR Revised Overt Aggression Scale OR Adapted Overt Aggression Scale).ti,ab.

16 Agitation Calmness Evaluation Scale.ti,ab.

17 (Cohen-Mansfield Agitation Inventory Observational OR CMAI-O).ti,ab.

18 Pittsburgh Agitation Scale.ti,ab.

19 (Positive and Negative Syndrome Scale Excited Component OR PANSS-EC).ti,ab.

20 (Ryden Aggression Scale-2 OR Ryden Aggression Scale OR Modified Ryden Aggression Scale OR Adapted Ryden Aggression Scale OR Revised Ryden Aggression Scale).ti,ab.

22 1 OR 2 OR 3 OR 4 OR 5 OR 6 OR 7 OR 8 OR 9 OR 10 OR 11 OR 12 OR 13 OR 14 OR 15 OR 16 OR 17 OR 18 OR 19 OR 20

AND

Psychometric

1. (instrumentation or methods).fs.

2. (Validation Studies or Comparative Study).pt.

3. exp Psychometrics/

4. psychometr*.ti,ab.

5. (clinimetr* or clinometr*).tw.

6. exp “Outcome Assessment (Health Care)”/

7. outcome assessment.ti,ab.

8. outcome measure*.tw.

9. exp Observer Variation/

10. observer variation.ti,ab.

11. exp Health Status Indicators/

12. exp “Reproducibility of Results”/

13. reproducib*.ti,ab.

14. exp Discriminant Analysis/

15. (reliab* or unreliab* or valid* or coefficient or homogeneity or homogeneous or “internal

consistency”).ti,ab.

16. (cronbach* and (alpha or alphas)).ti,ab.

17. (item and (correlation* or selection* or reduction*)).ti,ab.

18. (agreement or precision or imprecision or “precise values” or test-retest).ti,ab.

19. (test and retest).ti,ab.

20. (reliab* and (test or retest)).ti,ab.

21. (stability or interrater or inter-rater or intrarater or intra-rater or intertester or inter-tester

or intratester or intra-tester or interobserver or inter-observer or intraobserver or intraobserver

or intertechnician or inter-technician or intratechnician or intra-technician or

interexaminer or inter-examiner or intraexaminer or intra-examiner or interassay or interassay

or intraassay or intra-assay or interindividual or inter-individual or intraindividual or

intra-individual or interparticipant or inter-participant or intraparticipant or intra-participant

or kappa or kappa’s or kappas or repeatab*).ti,ab.

22. ((replicab* or repeated) and (measure or measures or findings or result or results or test or

tests)).ti,ab.

23. (generaliza* or generalisa* or concordance).ti,ab.

24. (intraclass and correlation*).ti,ab.

25. (discriminative or “known group” or factor analysis or factor analyses or dimension* or

subscale*).ti,ab.

26. (multitrait and scaling and (analysis or analyses)).ti,ab.

27. (item discriminant or interscale correlation* or error or errors or “individual

variability”).ti,ab.

28. (variability and (analysis or values)).ti,ab.

29. (uncertainty and (measurement or measuring)).ti,ab.

30. (“standard error of measurement” or sensitiv* or responsive*).ti,ab.

31. ((minimal or minimally or clinical or clinically) and (important or significant or detectable)

and (change or difference)).ti,ab.

32. (small* and (real or detectable) and (change or difference)).ti,ab.

33. (meaningful change or “ceiling effect” or “floor effect” or “Item response model” or IRT or

Rasch or “Differential item functioning” or DIF or “computer adaptive testing” or “item bank”

or “cross-cultural equivalence”).ti,ab.

34. 1 or 2 or 3 or 4 or 5 or 6 or 7 or 8 or 9 or 10 or 11 or 12 or 13 or 14 or 15 or 16 or 17 or 18 or

19 or 20 or 21 or 22 or 23 or 24 or 25 or 26 or 27 or 28 or 29 or 30 or 31 or 32 or 33

*PsychINFO*

1 “Agitated Behavior Scale” OR “Agitated Behaviour Scale”

2 “Rating Scale for Aggressive Behavior in the Elderly” OR “Rating Scale for Aggressive Behaviour in the Elderly”

3 “Staff Observation Aggression Scale”

4 “Aggressive Behavior Scale” OR “Aggressive Behaviour Scale”

5 “Brief Agitation Rating Scale”

6 “Cohen-Mansfield Agitation Inventory Short Form” OR “Short Form Cohen-Mansfield Agitation Inventory” OR “CMAI-SF”

7 “Overt Aggression Scale”

8 “Overt Aggression Scale-Modified for Neurorehabilitation” OR “OAS-MNR”

9 “Cohen-Mansfield Agitation Inventory” OR “Cohen-Mansfield Agitation” OR “CMAI”

10 “Clinical Global Impression Scale for Aggression” OR “CGI-A”

11 “Overt Agitation Severity Scale”

12 “Resistiveness to Care Scale”

13 “Scale for Observation of Agitation in Persons with Dementia of the Alzheimer type” OR “SOAPD”

14 “Staff Observation Aggression Scale-Revised OR Revised Staff Observation Aggression Scale” OR “Modified Observation Aggression Scale OR Adapted Observation Aggression Scale” OR “SOAS-R”

15 “Modified Overt Aggression Scale” OR “Revised Overt Aggression Scale” OR “Adapted Overt Aggression Scale”

16 “Agitation Calmness Evaluation Scale”

17 “Cohen-Mansfield Agitation Inventory Observational” OR “CMAI-O”

18 “Pittsburgh Agitation Scale”

19 “Positive and Negative Syndrome Scale Excited Component” OR “PANSS-EC”

20 “Ryden Aggression Scale-2” OR “Ryden Aggression Scale” OR “Modified Ryden Aggression Scale” OR “Adapted Ryden Aggression Scale” OR “Revised Ryden Aggression Scale”

22 1 OR 2 OR 3 OR 4 OR 5 OR 6 OR 7 OR 8 OR 9 OR 10 OR 11 OR 12 OR 13 OR 14 OR 15 OR 16 OR 17 OR 18 OR 19 OR 20

AND

1. exp Psychometrics/

2. psychometr*.ti,ab.

5. (clinimetr* or clinometr*).tw.

6. exp Health Outcomes/

7. outcome assessment.ti,ab.

8. outcome measure*.tw.

9. exp Interobserver Reliability/

10. observer variation.ti,ab.

11. Experimental Replication/

12. reproducib*.ti,ab.

14. exp Discriminant Validity/

15. (reliab* or unreliab* or valid* or coefficient or homogeneity or homogeneous or “internal

consistency”).ti,ab.

16. (cronbach* and (alpha or alphas)).ti,ab.

17. (item and (correlation* or selection* or reduction*)).ti,ab.

18. (agreement or precision or imprecision or “precise values” or test-retest).ti,ab.

19. (test and retest).ti,ab.

20. (reliab* and (test or retest)).ti,ab.

21. (stability or interrater or inter-rater or intrarater or intra-rater or intertester or inter-tester

or intratester or intra-tester or interobserver or inter-observer or intraobserver or intraobserver

or intertechnician or inter-technician or intratechnician or intra-technician or

interexaminer or inter-examiner or intraexaminer or intra-examiner or interassay or interassay

or intraassay or intra-assay or interindividual or inter-individual or intraindividual or

intra-individual or interparticipant or inter-participant or intraparticipant or intra-participant

or kappa or kappa’s or kappas or repeatab*).ti,ab.

22. ((replicab* or repeated) and (measure or measures or findings or result or results or test or

tests)).ti,ab.

23. (generaliza* or generalisa* or concordance).ti,ab.

24. (intraclass and correlation*).ti,ab.

25. (discriminative or “known group” or factor analysis or factor analyses or dimension* or

subscale*).ti,ab.

26. (multitrait and scaling and (analysis or analyses)).ti,ab.

27. (item discriminant or interscale correlation* or error or errors or “individual

variability”).ti,ab.

28. (variability and (analysis or values)).ti,ab.

29. (uncertainty and (measurement or measuring)).ti,ab.

30. (“standard error of measurement” or sensitiv* or responsive*).ti,ab.

31. ((minimal or minimally or clinical or clinically) and (important or significant or detectable)

and (change or difference)).ti,ab.

32. (small* and (real or detectable) and (change or difference)).ti,ab.

33. (meaningful change or “ceiling effect” or “floor effect” or “Item response model” or IRT or

Rasch or “Differential item functioning” or DIF or “computer adaptive testing” or “item bank”

or “cross-cultural equivalence”).ti,ab.

34. 1 or 2 or 3 or 4 or 5 or 6 or 7 or 8 or 9 or 10 or 11 or 12 or 13 or 14 or 15 or 16 or 17 or 18 or

19 or 20 or 21 or 22 or 23 or 24 or 25 or 26 or 27 or 28 or 29 or 30 or 31 or 32 or 33

*EMBASE*

1 “Agitated Behavior Scale” OR “Agitated Behaviour Scale”

2 “Rating Scale for Aggressive Behavior in the Elderly” OR “Rating Scale for Aggressive Behaviour in the Elderly”

3 “Staff Observation Aggression Scale”

4 “Aggressive Behavior Scale” OR “Aggressive Behaviour Scale”

5 “Brief Agitation Rating Scale”

6 “Cohen-Mansfield Agitation Inventory Short Form” OR “Short Form Cohen-Mansfield Agitation Inventory” OR “CMAI-SF”

7 “Overt Aggression Scale”

8 “Overt Aggression Scale-Modified for Neurorehabilitation” OR “OAS-MNR”

9 “Cohen-Mansfield Agitation Inventory” OR “Cohen-Mansfield Agitation” OR “CMAI”

10 “Clinical Global Impression Scale for Aggression” OR “CGI-A”

11 “Overt Agitation Severity Scale”

12 “Resistiveness to Care Scale”

13 “Scale for Observation of Agitation in Persons with Dementia of the Alzheimer type” OR “SOAPD”

14 “Staff Observation Aggression Scale-Revised” OR “Revised Staff Observation Aggression Scale” OR “Modified Observation Aggression Scale OR Adapted Observation Aggression Scale” OR “SOAS-R”

15 “Modified Overt Aggression Scale” OR “Revised Overt Aggression Scale” OR “Adapted Overt Aggression Scale”

16 “Agitation Calmness Evaluation Scale”

17 “Cohen-Mansfield Agitation Inventory Observational” OR “CMAI-O”

18 “Pittsburgh Agitation Scale”

19 “Positive and Negative Syndrome Scale Excited Component” OR “PANSS-EC”

20 “Ryden Aggression Scale-2” OR “Ryden Aggression Scale” OR “Modified Ryden Aggression Scale” OR “Adapted Ryden Aggression Scale” OR “Revised Ryden Aggression Scale”

22 1 OR 2 OR 3 OR 4 OR 5 OR 6 OR 7 OR 8 OR 9 OR 10 OR 11 OR 12 OR 13 OR 14 OR 15 OR 16 OR 17 OR 18 OR 19 OR 20

AND

1. exp Psychometrics/

2. psychometr*.ti,ab.

5. (clinimetr* or clinometr*).tw.

6. exp Outcome Assessment/

7. outcome assessment.ti,ab.

8. outcome measure*.tw.

9. exp Interobserver Reliability/

10. observer variation.ti,ab.

11. expl Replication study/

12. reproducib*.ti,ab.

14. exp Discriminant Validity/

15. (reliab* or unreliab* or valid* or coefficient or homogeneity or homogeneous or “internal

consistency”).ti,ab.

16. (cronbach* and (alpha or alphas)).ti,ab.

17. (item and (correlation* or selection* or reduction*)).ti,ab.

18. (agreement or precision or imprecision or “precise values” or test-retest).ti,ab.

19. (test and retest).ti,ab.

20. (reliab* and (test or retest)).ti,ab.

21. (stability or interrater or inter-rater or intrarater or intra-rater or intertester or inter-tester

or intratester or intra-tester or interobserver or inter-observer or intraobserver or intraobserver

or intertechnician or inter-technician or intratechnician or intra-technician or

interexaminer or inter-examiner or intraexaminer or intra-examiner or interassay or interassay

or intraassay or intra-assay or interindividual or inter-individual or intraindividual or

intra-individual or interparticipant or inter-participant or intraparticipant or intra-participant

or kappa or kappa’s or kappas or repeatab*).ti,ab.

22. ((replicab* or repeated) and (measure or measures or findings or result or results or test or

tests)).ti,ab.

23. (generaliza* or generalisa* or concordance).ti,ab.

24. (intraclass and correlation*).ti,ab.

25. (discriminative or “known group” or factor analysis or factor analyses or dimension* or

subscale*).ti,ab.

26. (multitrait and scaling and (analysis or analyses)).ti,ab.

27. (item discriminant or interscale correlation* or error or errors or “individual

variability”).ti,ab.

28. (variability and (analysis or values)).ti,ab.

29. (uncertainty and (measurement or measuring)).ti,ab.

30. (“standard error of measurement” or sensitiv* or responsive*).ti,ab.

31. ((minimal or minimally or clinical or clinically) and (important or significant or detectable)

and (change or difference)).ti,ab.

32. (small* and (real or detectable) and (change or difference)).ti,ab.

33. (meaningful change or “ceiling effect” or “floor effect” or “Item response model” or IRT or

Rasch or “Differential item functioning” or DIF or “computer adaptive testing” or “item bank”

or “cross-cultural equivalence”).ti,ab.

34. 1 or 2 or 3 or 4 or 5 or 6 or 7 or 8 or 9 or 10 or 11 or 12 or 13 or 14 or 15 or 16 or 17 or 18 or

19 or 20 or 21 or 22 or 23 or 24 or 25 or 26 or 27 or 28 or 29 or 30 or 31 or 32 or 33

**To identify studies that were conducted in patients with alcohol use disorder (AUD)**

*MEDLINE*

1 (Agitated Behavior Scale OR Agitated Behaviour Scale).ti,ab.

2 (Rating Scale for Aggressive Behavior in the Elderly OR Rating Scale for Aggressive Behaviour in the Elderly).ti,ab.

3 Staff Observation Aggression Scale.ti,ab.

4 (Aggressive Behavior Scale OR Aggressive Behaviour Scale).ti,ab.

5 Brief Agitation Rating Scale.ti,ab.

6 (Cohen-Mansfield Agitation Inventory Short Form OR Short Form Cohen-Mansfield Agitation Inventory OR CMAI-SF).ti,ab.

7 Overt Aggression Scale.ti,ab.

8 (Overt Aggression Scale-Modified for Neurorehabilitation OR OAS-MNR).ti,ab.

9 (Cohen-Mansfield Agitation Inventory OR Cohen-Mansfield Agitation OR CMAI).ti,ab.

10 (Clinical Global Impression Scale for Aggression OR CGI-A).ti,ab.

11 Overt Agitation Severity Scale.ti,ab.

12 Resistiveness to Care Scale.ti,ab.

13 (Scale for Observation of Agitation in Persons with Dementia of the Alzheimer type OR SOAPD).ti,ab.

14 (Staff Observation Aggression Scale-Revised OR Revised Staff Observation Aggression Scale OR Modified Observation Aggression Scale OR Adapted Observation Aggression Scale OR SOAS-R).ti,ab.

15 (Modified Overt Aggression Scale OR Revised Overt Aggression Scale OR Adapted Overt Aggression Scale).ti,ab.

16 Agitation Calmness Evaluation Scale.ti,ab.

17 (Cohen-Mansfield Agitation Inventory Observational OR CMAI-O).ti,ab.

18 Pittsburgh Agitation Scale.ti,ab.

19 (Positive and Negative Syndrome Scale Excited Component OR PANSS-EC).ti,ab.

20 (Ryden Aggression Scale-2 OR Ryden Aggression Scale OR Modified Ryden Aggression Scale OR Adapted Ryden Aggression Scale OR Revised Ryden Aggression Scale).ti,ab.

22 1 OR 2 OR 3 OR 4 OR 5 OR 6 OR 7 OR 8 OR 9 OR 10 OR 11 OR 12 OR 13 OR 14 OR 15 OR 16 OR 17 OR 18 OR 19 OR 20

AND

1 Wernicke-Korsakoff.ti,ab.

2 Korsakoff*.ti,ab.

3 Alcohol amnestic disorder.ti,ab.

4 Alcohol dementia.ti,ab.

5 alcohol*.ti,ab.

6 exp Alcoholism/

7 exp Korsakoff Syndrome/

8 exp Wernicke Encephalopathy/

9 exp Alcohol Amnestic Disorder/

10 exp Alcohol Abuse/

11 exp Alcohol Dependence/

12 1 OR 2 OR 3 OR 4 OR 5 OR 6 OR 7 OR 8 OR 9 OR 10 OR 11

*PsychINFO*

1 “Agitated Behavior Scale” OR “Agitated Behaviour Scale”

2 “Rating Scale for Aggressive Behavior in the Elderly” OR “Rating Scale for Aggressive Behaviour in the Elderly”

3 “Staff Observation Aggression Scale”

4 “Aggressive Behavior Scale” OR “Aggressive Behaviour Scale”

5 “Brief Agitation Rating Scale”

6 “Cohen-Mansfield Agitation Inventory Short Form” OR “Short Form Cohen-Mansfield Agitation Inventory” OR “CMAI-SF”

7 “Overt Aggression Scale”

8 “Overt Aggression Scale-Modified for Neurorehabilitation” OR “OAS-MNR”

9 “Cohen-Mansfield Agitation Inventory” OR “Cohen-Mansfield Agitation” OR “CMAI”

10 “Clinical Global Impression Scale for Aggression” OR “CGI-A”

11 “Overt Agitation Severity Scale”

12 “Resistiveness to Care Scale”

13 “Scale for Observation of Agitation in Persons with Dementia of the Alzheimer type” OR “SOAPD”

14 “Staff Observation Aggression Scale-Revised OR Revised Staff Observation Aggression Scale” OR “Modified Observation Aggression Scale OR Adapted Observation Aggression Scale” OR “SOAS-R”

15 “Modified Overt Aggression Scale” OR “Revised Overt Aggression Scale” OR “Adapted Overt Aggression Scale”

16 “Agitation Calmness Evaluation Scale”

17 “Cohen-Mansfield Agitation Inventory Observational” OR “CMAI-O”

18 “Pittsburgh Agitation Scale”

19 “Positive and Negative Syndrome Scale Excited Component” OR “PANSS-EC”

20 “Ryden Aggression Scale-2” OR “Ryden Aggression Scale” OR “Modified Ryden Aggression Scale” OR “Adapted Ryden Aggression Scale” OR “Revised Ryden Aggression Scale”

22 1 OR 2 OR 3 OR 4 OR 5 OR 6 OR 7 OR 8 OR 9 OR 10 OR 11 OR 12 OR 13 OR 14 OR 15 OR 16 OR 17 OR 18 OR 19 OR 20

AND

1 “Wernicke-Korsakoff”

2 “Korsakoff*”

3 “Alcohol amnestic disorder”

4 “Alcohol dementia”

5 “alcohol*”

6 exp Alcoholism/

7 exp Korsakoff Syndrome/

8 exp Wernicke Encephalopathy/

9 exp Alcohol Amnestic Disorder/

10 exp Alcohol Abuse/

11 exp Alcohol Dependence/

12 1 OR 2 OR 3 OR 4 OR 5 OR 6 OR 7 OR 8 OR 9 OR 10 OR 11

*EMBASE*

1 “Agitated Behavior Scale” OR “Agitated Behaviour Scale”

2 “Rating Scale for Aggressive Behavior in the Elderly” OR “Rating Scale for Aggressive Behaviour in the Elderly”

3 “Staff Observation Aggression Scale”

4 “Aggressive Behavior Scale” OR “Aggressive Behaviour Scale”

5 “Brief Agitation Rating Scale”

6 “Cohen-Mansfield Agitation Inventory Short Form” OR “Short Form Cohen-Mansfield Agitation Inventory” OR “CMAI-SF”

7 “Overt Aggression Scale”

8 “Overt Aggression Scale-Modified for Neurorehabilitation” OR “OAS-MNR”

9 “Cohen-Mansfield Agitation Inventory” OR “Cohen-Mansfield Agitation” OR “CMAI”

10 “Clinical Global Impression Scale for Aggression” OR “CGI-A”

11 “Overt Agitation Severity Scale”

12 “Resistiveness to Care Scale”

13 “Scale for Observation of Agitation in Persons with Dementia of the Alzheimer type” OR “SOAPD”

14 “Staff Observation Aggression Scale-Revised” OR “Revised Staff Observation Aggression Scale” OR “Modified Observation Aggression Scale OR Adapted Observation Aggression Scale” OR “SOAS-R”

15 “Modified Overt Aggression Scale” OR “Revised Overt Aggression Scale” OR “Adapted Overt Aggression Scale”

16 “Agitation Calmness Evaluation Scale”

17 “Cohen-Mansfield Agitation Inventory Observational” OR “CMAI-O”

18 “Pittsburgh Agitation Scale”

19 “Positive and Negative Syndrome Scale Excited Component” OR “PANSS-EC”

20 “Ryden Aggression Scale-2” OR “Ryden Aggression Scale” OR “Modified Ryden Aggression Scale” OR “Adapted Ryden Aggression Scale” OR “Revised Ryden Aggression Scale”

22 1 OR 2 OR 3 OR 4 OR 5 OR 6 OR 7 OR 8 OR 9 OR 10 OR 11 OR 12 OR 13 OR 14 OR 15 OR 16 OR 17 OR 18 OR 19 OR 20

AND

1 “Wernicke-Korsakoff”

2 “Korsakoff*”

3 “Alcohol amnestic disorder”

4 “Alcohol dementia”

5 “alcohol*”

6 exp Alcoholism/

7 exp Korsakoff Syndrome/

8 exp Wernicke Encephalopathy/

9 exp Alcohol Amnestic Disorder/

10 exp Alcohol Abuse/

11 exp Alcohol Dependence/

13-06-2024: n=835568

**Supplemental Material: Scales included and excluded in review**

| **Scale identified through search** | **Reason to exclude** | | | | **Included in review** |
| --- | --- | --- | --- | --- | --- |
|  | **Composite scale** | **Predicts future behavior** | **Outpatient setting** | **Self-report** |  |
| Aggressive Behavior Risk Assessment Tool for Long-Term Care |  | X |  |  |  |
| Aggressive Behavior Scale |  |  |  |  | X |
| Agitated Behavior in Dementia scale |  |  | X |  |  |
| Agitated Behavior Scale |  |  |  |  | X |
| Agitation Calmness Evaluation Scale |  |  |  |  | X |
| Agitation Severity Scale |  |  | X |  |  |
| Alzheimer's Disease Assessment Scale non-cog | X |  |  |  |  |
| Behavioral and Mood Disturbance Scale | X |  |  |  |  |
| Behavioral Pathology in Alzheimer’s Disease | X |  |  |  |  |
| Brief Agitation Rating Scale |  |  |  |  | X |
| Brief Psychiatric Rating Scale | X |  |  |  |  |
| Brøset Violence Checklist |  | X |  |  |  |
| Buss-Perry Aggression Questionnaire |  |  |  | X |  |
| Buss-Perry Aggression Questionnaire |  |  |  | X |  |
| Clinical Global Impression Scale for Aggression |  |  |  |  | X |
| Cohen-Mansfield Agitation Inventory Community version |  |  | X |  |  |
| Cohen-Mansfield Agitation Inventory Observational tool |  |  |  |  | X |
| Cohen-Mansfield Agitation Inventory Short Form |  |  |  |  | X |
| Columbia University Scale for Psychopathology in Alzheimer’s Disease | X |  |  |  |  |
| Comprehensive PsychopathologicalRating Scale | X |  |  |  |  |
| Conflicts Tactics Scale |  |  |  | X |  |
| Dementia Behavior Disturbance scale | X |  |  |  |  |
| Disruptive Behavior Rating Scale | X |  |  |  |  |
| Dynamic Appraisal of Situational Aggression Inpatient Version |  | X |  |  |  |
| Emotions Profile Index | X |  |  |  |  |
| Frontal System Behavior Scale | X |  |  |  |  |
| Geriatric Mental State Schedule | X |  |  |  |  |
| Historical, Clinical, Risk Management-20 |  | X |  |  |  |
| Minnesota Multiphasic Personality Inventory-2 | X |  |  |  |  |
| Modified Overt Aggression Scale |  |  |  |  | X |
| Multi-Dimensional Dementia Assessment Scale | X |  |  |  |  |
| Neurobehavioral Rating Scale | X |  |  |  |  |
| Neuropsychiatric Inventory | X |  |  |  |  |
| Nurses' Scale for In-patient Evaluation | X |  |  |  |  |
| Overt Aggression Scale |  |  |  |  | X |
| Overt Aggression Scale Modified |  |  |  |  | X |
| Overt Aggression Scale Modified for Neurorehabilitation |  |  |  |  | X |
| Overt Agitation Severity Scale |  |  |  |  | X |
| Patient-Staff Conflict Checklist | X |  |  |  |  |
| Pittsburgh Agitation Scale |  |  |  |  | X |
| Positive and Negative Syndrome Scale | X |  |  |  |  |
| Positive and Negative Syndrome Scale Excited Component Excited Component |  |  |  |  | X |
| Present State Examination | X |  |  |  |  |
| Rating Scale for Aggressive Behavior in the Elderly |  |  |  |  | X |
| Report Form for Aggressive episodes |  |  | X |  |  |
| Resistiveness to Care Scale |  |  |  |  | X |
| Ryden Aggression Scale-2 |  |  |  |  | X |
| Scale for Observation of Agitation in Persons with Dementia of the Alzheimer type |  |  |  |  | X |
| Short-Term Assessment of Risk and Treatability |  | X |  |  |  |
| St. Andrews-Swansea Neurobehavioural Outcome Scale | X |  |  |  |  |
| Staff Observation Aggression Scale |  |  |  |  | X |
| Staff Observation Aggression Scale-Revised |  |  |  |  | X |
| The Behavior Rating Scale for Dementia | X |  |  |  |  |
| The McNiel-Binder Violence Screening Checklist |  | X |  |  |  |
| The Nursing Home Behavior Problem Scale | X |  |  |  |  |
| Trauma Complaints List | X |  |  |  |  |
